# Animal models for the study of hepatitis B virus infection

**DOI:** 10.24272/j.issn.2095-8137.2018.013

**Published:** 2018-02-09

**Authors:** Wei-Na Guo, Bin Zhu, Ling Ai, Dong-Liang Yang, Bao-Ju Wang

**Affiliations:** 1 Department of Infectious Diseases, Union Hospital, Tongji Medical College, Huazhong University of Science and Technology, Wuhan Hubei 430022, China

**Keywords:** Hepatitis B virus, Animal model, Duck hepatitis B virus, Woodchuck hepatitis virus

## Abstract

Even with an effective vaccine, an estimated 240 million people are chronically infected with hepatitis B virus (HBV) worldwide. Current antiviral therapies, including interferon and nucleot(s)ide analogues, rarely cure chronic hepatitis B. Animal models are very crucial for understanding the pathogenesis of chronic hepatitis B and developing new therapeutic drugs or strategies. HBV can only infect humans and chimpanzees, with the use of chimpanzees in HBV research strongly restricted. Thus, most advances in HBV research have been gained using mouse models with HBV replication or infection or models with HBV-related hepadnaviral infection. This review summarizes the animal models currently available for the study of HBV infection.

## INTRODUCTION

One-third of the world’s population have experienced hepatitis B virus (HBV) infection, with 240 million people chronically infected. Although hepatitis B vaccination programs have greatly reduced HBV infection rates in adolescents in China, there are still 93 million chronic HBV carriers (Chinese Society of Hepatology, Chinese Medical Association & Chinese Society of Infectious Diseases, Chinese Medical Association, 2015). Existing drug interferon and nucleoside (acid) analogues have low response rates and cannot effectively remove HBV; therefore, exploring new drugs or treatment programs is still an urgent problem in the field of HBV research.

Animal models are promising tools for solving the above issue; however, such models are currently hampered because of the extremely narrow host range. HBV is the prototypic member of the Hepadnaviridae family, which contains closely-related hepatotropic, enveloped DNA viruses, including HBV, duck hepatitis B virus (DHBV), and woodchuck hepatitis virus (WHV). Chimpanzees are the only non-human primate fully susceptible to HBV infection, though their use in HBV research is strongly restricted (Wieland, 2015). Several non-primate species can be infected with HBV, including the tree shrew (*Tupaia belangeri*), which also exhibits a close phylogenetic relationship with primates (Xiao et al., 2017). In addition, primary *Tupaia* hepatocytes are suitable for HBV study, with sodium taurocholate co-transporting polypeptide (NTCP) recently identified using this system (Yan et al., 2012). Animals with non-HBV hepadnaviral infection have also been used in HBV research for many decades. Though DHBV is distantly related to HBV, primary duck hepatocytes and ducklings are easily available and have made great contribution in elucidating the unique replication mechanism of hepadnaviruses and in evaluating antiviral drugs (Mason, 2015). Furthermore, WHV infection in newborn American woodchucks (*Marmota monax*) can led to persistent infection and hepatocellular carcinoma (HCC) (Menne & Cote, 2007). Recently, we found the Chinese woodchuck (*Marmota himalayana*) to be highly susceptible to WHV infection (Wang et al., 2011). Woodchucks are widely used in exploring the pathogenesis of chronic hepadnavirus infection and HCC development, as well as in assessing antiviral drugs and immunotherapeutic approaches. Although the mouse is the best characterized and most convenient laboratory animal, it cannot be infected with HBV. However, after considerable effort over many decades, several mouse models with HBV replication and chimeric mouse models harboring human hepatocytes with HBV infection have been successfully established. In this review, we summarize the animal models currently available for the study of HBV infection.

## ANIMAL MODELS WITH HBV INFECTION

### Chimpanzee model

The chimpanzee is the only immunocompetent animal fully susceptible to HBV infection. After inoculation with serum from HBV patients, chimpanzees can develop acute and chronic HBV infection, accompanied by similar liver inflammation and cellular immune responses as patients with HBV infection (Wieland, 2015), which closely mimics the natural history of HBV infection in humans (Table 1).

**Table 1:** Characteristics of animal models for HBV study

	Virological characteristics				Animal characteristics			References
	Virus	Viral entry	Viral infection/replication	cccDNA formation	Immunocompetent/immunodeficient	Inbred	Class/Order/Family/Genus	
Chimpanzee	HBV	Yes	Infection	Yes	Immunocompetent	No	Mammalia/Primate/Hominidae/ *Pan*	Wieland, 2015
*Tupaia*	HBV	Yes	Infection	Yes	Immunocompetent	No	Mammalia/Scandentia/Tupaiidae/ *Tupaia*	Xiao et al., 2017; Yan et al., 2012; Yang et al., 2015; Wang et al., 2012
Human chimeric mouse	HBV	Yes	Infection	Yes	Immunodeficient	Yes	Mammalia/Rodentia/Muridae/ *Mus*	Bissig et al., 2010; Dandri et al., 2001; Tsuge et al., 2005
Duck	DHBV	Yes	Infection	Yes	Immunocompetent	No	Aves/Anseriformes/Anatidae	Jilbert et al., 1996; Mason, 2015; Schultz et al., 2004
Woodchuck	WHV	Yes	Infection	Yes	Immunocompetent	No	Mammalia/Rodentia/Sciuridae/ *Marmota*	Cote et al., 2000; Menne & Cote, 2007; Roggendorf et al., 2007; Wang et al., 2011
HBV transgenic mouse model	HBV	No	Replication	No	Immunocompetent, tolerant to HBV	Yes	Mammalia/Rodentia/Muridae/ *Mus*	Guidotti et al., 1995
HBV transfected mouse model	HBV	No	Replication	No	Immunocompetent	Yes	Mammalia/Rodentia/Muridae/ *Mus*	Huang et al., 2006, 2012; Peng et al., 2015; Yang et al., 2002

Chimpanzee models are essential for evaluating the safety and efficacy of HBV vaccines (McAuliffe et al., 1980), and have also been used to assess HBV therapeutic strategies (Eren et al., 2000). Recent studies have found that GS-9620, an oral agonist of toll-like receptor-7, can effectively reduce HBV titers in chimpanzees by inducing NK cell and virus-specific T cell responses (Lanford et al., 2013). Chimpanzees have also been used for exploring the pathogenesis of HBV infection. Using this model, a strong and polyclonal CD8^+^ T cell response against HBV was determined to be a key factor in viral clearance through cytopathic and non-cytopathic pathways, with CD4^+^ T cell priming crucial in the early phase of HBV infection (Asabe et al., 2009; Thimme et al., 2003).

However, due to the ethical and availability limitations, chimpanzees are not routinely used in HBV research. Continuous efforts have been made to establish HBV infection in smaller non-human primates. Recently, cynomolgus monkeys from Mauritius were found with naturally occurring transmissible chronic HBV infection (Dupinay et al., 2013), and rhesus macaques can establish HBV infection after viral vector-mediated *in vivo* expression of human NTCP in its hepatocytes (Burwitz et al., 2017).

### *Tupaia* model

The tree shrew (*Tupaia belangeri*) belongs to Tupaiidae, Scandentia, Mammalia, and displays a close phylogenetic relationship with primates. Recently, the whole genome sequence of the Chinese tree shrew implied that the nervous, immune, and metabolic systems were similar to those of humans (Xiao et al., 2017). To date, the tree shrew is the only non-primate animal found to be susceptible to HBV infection. Primary *Tupaia* hepatocytes are easily available compared with primary human hepatocytes, and therefore have been used in HBV research for many years (Table 1). NTCP, which is the cellular receptor responsible for HBV entry, was recently identified in the *Tupaia* model (Yan et al., 2012). Although HBV infection in neonatal tree shrews can lead to chronicity and pathological changes, including fibrosis, *in vivo* infection efficiency in *Tupaia* needs improvement (Wang et al., 2012; Yang et al., 2015). Recently, genotype A2 HBV isolates in *Tupaia belangeri* from Japan were found with higher HBV replication and chronicity rates, with the interferon response found to be impaired by HBV infection (Kayesh et al., 2017).

### Human chimeric mice

The first human liver chimeric mouse model was developed in immunodeficient (Rag2-/-, SCID, SCID/beige) mice with the urokinase-type plasminogen activator (uPA) transgene. The expression of the uPA gene can induce necrosis of hepatocytes, leading to subacute liver failure in young mice, and making it possible to transplant human hepatocytes into mouse livers. Transplantation of human hepatocytes into uPA-SCID mice results in a liver-humanized model with high human hepatocyte reconstitution rate and supportive of HBV and HCV infection (Dandri et al., 2001; Tsuge et al., 2005) (Table 1).

A chimeric mouse model was constructed using FRG (*Fah^-/-^/Rag2^-/-^/Il2rg^-/-^*) mice, in which liver failure can be induced at will (Azuma et al., 2007). Results showed that fumarylacetoacetate hydrolase (Fah) deficiency led to liver failure, and double knock out of Rag2 and the gamma chain of the IL-2 receptor resulted in sufficient human liver cell repopulation in mice. Administration of 2-(2-nitro-4-trifluoro-methylbenzoyl)-1,3-cyclohexanedione (NTBC) protected FRG mice from liver injury until NTBC withdrawal. Subsequent improvements, including refining transplantation procedures and increasing numbers of transplanted cells, increased liver cell repopulation rates to 95% in FRG mice (Bissig et al., 2010). Human liver cells repopulated in FRG mice support HBV and hepatitis C virus (HCV) infection (Table 1).

The chimeric mouse model can be used for investigations on the biology of HBV infections, not only the distinct HBV genotypes but also naturally occurring variants or drug-resistant mutants. This model has also been used for studying interactions of HBV with the host and in testing novel antivirals. A new monoclonal antibody targeting a unique HBV surface antigen (HBsAg) epitope has been found to induce prolonged suppression of HBV in human hepatocytes transplanted into FRG mice (Zhang et al., 2016). Furthermore, NVR3–778, a novel capsid assembly modulator, was found to have high antiviral activity by reducing serum HBV DNA and HBV RNA levels in uPA/SCID mice with humanized livers (Klumpp et al., 2017). Two ribonuclease H inhibitors, α-hydroxytropolone and N-hydroxypyridinedione, have been found to suppress HBV replication in FRG human liver chimeric mice (Long et al., 2018).

Because chimeric animals are genetically immunodeficient, they are unsuitable for the study of adaptive immunity or immunotherapy strategies. Researchers have used a variety of strategies to develop chimeric mice dually reconstituted with both immune cells and hepatocytes of human origin, including AFC8 (Washburn et al., 2011) and A2/NSG (Bility et al., 2014) mouse models. The former carries combined Rag2-/-/Il2rg-/- immunodeficiency genes, whereas the latter consists of NOD-SCID Il2rg-/- immunodeficient mice harboring the human HLA-A2 gene. Liver injury in both mouse models can be induced with inducible caspase-8 expression and CD95-activating antibodies, respectively. Liver cells and immune precursor cells from human fetal liver tissue have been transplanted into these two mouse models, with the AFC8 model used to study HCV infection (Washburn et al., 2011) and the A2/NSG model shown to support persistent HBV infection, accompanied by liver inflammation and fibrosis (Bility et al., 2014) (Table 1).

## ANIMAL MODELS WITH NON-HBV HEPADNAVIRAL INFECTION

### Duck model

Mason et al. (1980) found a DNA virus similar to human HBV in the serum of ducks, named DHBV (Table 1) and subsequently classified as *Avihepadnavirus*. Similar to HBV infection in humans, age and inoculum dose can influence the outcome of DHBV infection. For example, 7-day-old ducklings can exhibit persistent infection when inoculated with high-titer DHBV inoculum (Cote et al., 2000). The rates of natural HBV infection can also vary in distinct duck species, ranging from 8.75% to 17.80%, and the rates of experimental DHBV infection can differ in ducklings inoculated via intraperitoneal or intravenous injection (65% vs. 80%) (Hao et al., 2012). Duck models with persistent DHBV infection are widely used to evaluate anti-HBV drugs and therapeutic strategies; for example nucleot(s)ide analogs commonly used in clinicals such as entacavir (ETV) (Marion et al., 2002), nucleocapsid assembly inhibitors (Campagna et al., 2013), nucleic acid polymer REP 2139 (Quinet et al., 2017), and combined immunotherapy (Le Guerhier et al., 2003). This animal model has also been used to study the pathogenesis of HBV infection. A lack of innate immunity rather than adaptive immunity in early phase (within 5 days after DHBV inoculation) DHBV infection is important in DHBV persistence in newborn ducks (Tohidi-Esfahani et al., 2010). Residual cccDNA pools are detectable and unchanged in ducks following resolution of acute HBV infection, even after ETV treatment (Reaiche et al., 2010). After continuous DNA vaccination combined with interleukin (IL)-2 and interferon (IFN)-γ plasmid injections, only trace cccDNA is detectable in duck liver, whereas inoculation of naïve ducks with these liver homogenates can cause high levels of DHBV infection (Saade et al., 2013). 

### Woodchuck model

In 1978, WHV was found in a colony of American woodchucks in Penrose Zoo, Philadelphia, with high rates of chronic hepatitis and HCC observed (Summers et al., 1978), and was subsequently identified as *Orthohepadnavirus*. WHV is very similar to HBV not only in terms of virological characteristics, but also the natural history of infection and immune response against viral infection (Cote et al., 2000) (Table 1). Therefore, it is widely used to study the pathogenesis of HBV infection (Fletcher et al., 2013; Menne et al., 1998), and to evaluate antiviral drugs, prophylactic vaccines, and immunotherapeutic strategies (Colonno et al., 2001; Kosinska et al., 2013; Roggendorf et al., 2007).

We found the Chinese woodchuck (*Marmota himalayana*), a relative of the American woodchuck, to be highly susceptible to WHV, suggesting potential as a new animal model for HBV infection (Wang et al., 2011). However, the lack of genomic information on the Chinese woodchuck has strongly limited its application in HBV research. Recently, however, the transcriptome sequences of this species have been fully sequenced and analyzed, with high sequence similarity found between the Chinese and American woodchucks (Liu et al., 2016). To improve the quality of the Chinese woodchuck model, we inoculated animals with different dosages of WHV inoculum (104, 106, or 108 genome equivalents of WHV7), and found that different doses had a strong influence on the course of WHV infection (Wang et al., 2014); we also inoculated animals from different counties (i.e., Tongren, Tongde, Guide, and Wulan), and found varied susceptibilities to WHV infection (unpublished data). For better application of the Chinese woodchuck model, breeding colonies have been established in Qinghai and Xinjiang in northwest China. Furthermore, over 20 genes from woodchucks have been analyzed, including IL-6 receptor, IL-12 receptor, IL-18 and its receptor, IL-21 receptor, tumor growth factor-βs and their receptors, CD40, CD160, CD244, Tim-3, galectin-9, retinoic acid-inducible gene I, melanoma differentiation-associated gene 5, AIM-2, IFI16, RelA, and interferon stimulated gene (Fan et al., 2012; Jiang et al., 2012; Lu et al., 2008; Wang et al., 2016; Yan et al., 2016; Yang et al., 2013). These efforts have improved the Chinese woodchuck model for application in HBV research. An alternative strategy for HBV post-exposure prophylaxis based on nucleotide analogues (NA) was evaluated using this model, with NA alone or in combination with a DNA vaccine found to completely prevent viremia after WHV inoculation, but induce partial or complete protective immunity, respectively (Wang et al., 2014).

## MOUSE MODEL SUPPORTING HBV REPLICATION

### HBV transgenic mouse model

Since the 1980s, HBV transgenic mice selectively expressing HBV proteins, including HBsAg, e antigen, or x protein, have been used to study the role of these proteins. In 1995, a transgenic mouse model for the full HBV genome was established, which produced infectious HBV virions morphologically indistinguishable from human-derived virions (Guidotti et al., 1995) (Table 1). Although HBV is immune-tolerant and cannot be spontaneously cleared as its genome is integrated in the mouse genome, this model has made important contributions in elucidating the pathogenesis of HBV infection: for example, no liver damage has been observed in HBV transgenic mice, suggesting that HBV itself is not cytopathic; and the adoptive transfer of HBV-specific cytotoxic T cells can lead to liver damage in HBV transgenic mice and transient clearance of HBsAg through lysis of HBsAg-expressing cells, whereas viral replication can also be suppressed by non-cytolytic mechanisms involving IFN-α and tumor necrosis factor-α (TNF-α) (Guidotti et al., 1996; Heise et al., 1999). HBV transgenic mice have also been used for testing antiviral drugs, such as lamivudine, adefovir dipivoxil, ETV, and HBV-targeting siRNAs (Ebert et al., 2011; McCaffrey et al., 2003). In China, HBV transgenic mice have also been established and applied in HBV research, including: (1) evaluating a DNA vaccine by fusion of the granulocyte-macrophage colony stimulating factor gene to the HBV-S gene (Qing et al., 2010); (2) analyzing hepatocyte proteomics and seroproteomics in HBV-transgenic mice (Ding et al., 2009); and (3) exploring the role of NK cells in CCL4 accelerated liver fibrosis (Jin et al., 2011).

### HBV transfected mouse model

Viral vectors based on adenoviruses or adeno-associated viruses containing HBV genomes efficiently transduce hepatocytes in immunocompetent mice. After intravenous injection of high doses of HBV containing adenoviral vectors (Ad-HBV), HBV replication lasts for only three months because transduction of ad-HBV often results in a strong immune response against the adenovirus itself. This can be overcome by the injection of low doses of Ad-HBV (Huang et al., 2012) (Table 1).

Injecting a large amount of liquid containing naked DNA into mice by the tail vein over a short period of time terms as hydrodynamic injection, which can effectively transfer exogenous genes into liver cells. Hydrodynamic injection was found to successfully deliver replication-competent HBV genomes into the livers of immunocompetent mice, with viremia peaking after 6 d and rapidly declining thereafter (Yang et al., 2002). It should be noted that plasmid backbone, mouse strain, and sex can affect HBV replication duration in mice. HBV replication was found to last for up to six months using the pAAV/HBV1.2 plasmid and C57BL/6 mice (Huang et al., 2006), and up to 46 weeks using the pAAV/HBV1.2 plasmid and C3H/HeN mice (Peng et al., 2015) (Table 1). This animal model can be used to analyze immune responses during acute or persistent HBV replication, clarify the biology of different HBV genotypes, variants, or mutants *in vivo*, and evaluate antiviral compounds. Using this model, we discovered that Poly (I: C) cleared HBV through IFN-dependent pathways (Wu et al., 2014). Recently, one genotype B HBV isolate, designated BPS, persisted for 33 weeks in ~50% of mice after hydrodynamic injection, with IL-21 and IL-33 found to be potent regulators of HBV clearance (Shen et al., 2017).

HBV cccDNA plays a major role in HBV persistence and is responsible for the relapse of viral activity after antiviral treatment with polymerase inhibitors in chronically infected patients, while is not built in not only HBV transgenic mice but also HBV transfected mice. To overcome this large obstacle and closely mimic chronic HBV infection in humans, Li et al. (2018) established a surrogate mouse model with HBV cccDNA persistence; using a replication-defective recombinant adenoviral vector, recombinant HBV cccDNA was successfully delivered into the mouse liver and was intrinsically stable, resulting in HBV persistence throughout the experiment (>62 weeks) in transgenic mice expressing Cre recombinase under the albumin promoter.

## SUMMARY

The above animal models with HBV replication or hepadnavirus infection provide more options for HBV research. The identification of NTCP as an HBV receptor is a milestone in the field of HBV research. The NTCP-transfected hepatoma cell line is the first human cell line supporting HBV infection, while NTCP transgenic mice support HBV infection was ongoing. Transfected, transduced, or transgenic mice with HBV genomes can only support HBV replication, with the HBV life cycle incomplete due to the lack of viral entry, cccDNA formation, and viral spread. As human liver cells supporting HBV infection are transplanted into immunodeficient mice, these mice exhibit obvious immunodeficiency and their maintenance systems are very complex. The chimpanzee is an optimal model for HBV infection, but is strongly restricted and not easily available for HBV research. Tree shrews can be infected with HBV, but the *in vivo* system requires considerable improvement. For the duck and woodchuck models, the differences between HBV and other hepadnaviruses should be considered. In addition, except for mice, the above models are not inbred and detection reagents are not readily available. Thus, all current animal models have specific limitations. Therefore, researchers need to carefully interpret their results from animal studies, and validation of their findings in multiple systems should be encouraged.
